# Dual modes of action: direct regulation and ceRNA-mediated mechanisms of ncRNAs in dermal fibroblast senescence

**DOI:** 10.3389/fcell.2026.1874939

**Published:** 2026-06-29

**Authors:** Juanlie Luo, Jiabao Ma, Hui Tian, Wenling Yang, Meizhen Wu, Xiaojiao Pan, Qingtong Lan

**Affiliations:** 1 School of Pharmacy, Guangxi University of Chinese Medicine, Nanning, China; 2 The First Affiliated Hospital of Guangxi University of Chinese Medicine, Nanning, China

**Keywords:** competing endogenous RNAs, dermal fibroblasts, direct regulation, dual regulatory mechanisms, non-coding RNAs, senescence

## Abstract

Skin aging is characterized by progressive decline in dermal fibroblast function, marked by reduced extracellular matrix (ECM) synthesis, accelerated ECM degradation, diminished repair capacity, and activation of the senescence-associated secretory phenotype (SASP). Recent studies demonstrate that non-coding RNAs (ncRNAs), including microRNAs (miRNAs), long non-coding RNAs (lncRNAs), and circular RNAs (circRNAs), critically regulate this process through two primary mechanisms. First, ncRNAs exert direct regulation, including miRNA-mediated RNA-RNA targeting and lncRNA/circRNA-mediated direct binding to proteins or DNA/chromatin. Second, certain lncRNAs and circRNAs act as competing endogenous RNAs (ceRNAs) by sequestering miRNAs, thereby derepressing miRNA-targeted genes. This review systematically outlines the dual regulatory roles of miRNAs, lncRNAs, and circRNAs in dermal fibroblast senescence, summarizes the ceRNA network features in replicative aging, photoaging, and stress-induced premature senescence, and highlights advances and experimental strategies for validating direct regulation. We also note current limitations, including imbalanced research focus on regulatory modes, unclear functions of emerging ncRNAs such as PIWI-interacting RNAs (piRNAs) and small nucleolar RNAs (snoRNAs), and insufficient characterization of fibroblast subpopulation heterogeneity. Future work should integrate single-cell and spatial omics to deepen mechanistic insights and develop ncRNA-based targeted delivery and precision anti-aging interventions.

## 1 Introduction

The skin is the largest organ of the human body, and its structural integrity and functional homeostasis are highly dependent on dermal fibroblasts. These cells are responsible for synthesizing extracellular matrix (ECM) components such as collagen and elastin, and modulate inflammatory responses (Zhang J. et al., 2024). However, during intrinsic aging or photoaging induced by chronic ultraviolet (UV) exposure, dermal fibroblasts gradually undergo progressive functional decline, characterized by reduced ECM synthesis, overexpression of matrix metalloproteinases (MMPs), activation of the senescence-associated secretory phenotype (SASP), and diminished regenerative capacity, ultimately leading to skin laxity, wrinkle formation, and impaired barrier function (Nishikori et al., 2023; Quan et al., 2023; Wang B. et al., 2024). In recent years, epigenetic and epitranscriptomic regulation has emerged as key mechanisms driving skin aging (Dela et al., 2025).

**FIGURE 1:**
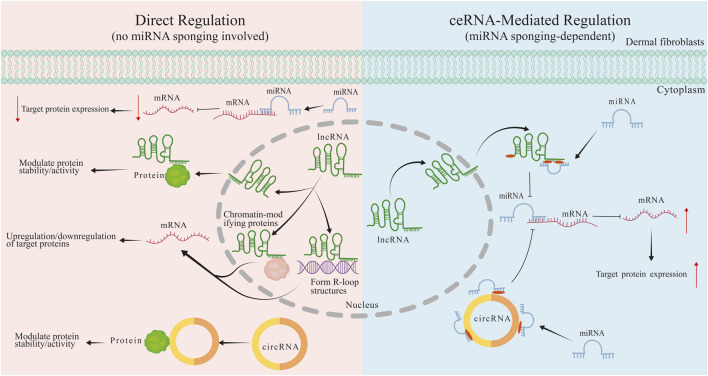
Two core regulatory modes of ncRNAs in dermal fibroblast senescence. NcRNAs modulate dermal fibroblast senescence through two independent regulatory mechanisms. Left panel: Direct regulation (no miRNA sponging involved). Right panel: ceRNA-mediated regulation (miRNA sponging-dependent).

Within this regulatory framework, non-coding RNAs (ncRNAs) play a central role, encompassing microRNAs (miRNAs), long non-coding RNAs (lncRNAs), and circular RNAs (circRNAs), among others (Roso-Mares et al., 2024). These ncRNAs exert pivotal functions in the multi-layered regulation of gene expression. Notably, ncRNAs do not function solely through a single pathway (Figure 1). They primarily operate through two core mechanisms. The first is direct regulation, which includes miRNA-mediated RNA-RNA targeting and lncRNA/circRNA-mediated direct binding to proteins or DNA/chromatin (Kim et al., 2017). The second is the competing endogenous RNA (ceRNA) mechanism, whereby certain lncRNAs or circRNAs act as molecular sponges that sequester miRNAs, thereby indirectly relieving the suppression of their target messenger RNAs (Gao et al., 2023; Zou et al., 2023). However, existing reviews predominantly explore the functions of ncRNAs in skin aging separately (Soheilifar et al., 2022) or focus on the construction of ceRNA networks (Kim, 2023), lacking a systematic distinction and integration of these two regulatory paradigms. Such a distinction holds significant scientific importance: misclassifying direct regulation as ceRNA mechanism, or vice versa, not only misleads the understanding of molecular mechanism but also impacts the design of targeted intervention strategies. For example, developing small-molecule drugs that interfere with the protein-binding domains of lncRNAs that directly bind to proteins represents a completely different technical approach compared to designing miRNA sponge inhibitors targeting ceRNAs (Watmuff et al., 2025). Therefore, this review aims to construct a clear analytical framework to distinguish these two regulatory modes and systematically elaborate the dual actions of ncRNAs during dermal fibroblast senescence. We also clarify the molecular characteristics, identification criteria and functional interactions between direct regulation and ceRNA-mediated regulation. Specifically, our main objectives are as follows: (1) to establish unified identification criteria for direct regulation and ceRNA regulation based on available experimental evidence; (2) to categorize the functions of miRNAs, lncRNAs and circRNAs across replicative aging, photoaging and stress-induced premature senescence according to the two regulatory patterns; (3) to discuss the dynamic features, network crosstalk and functional antagonism of ncRNA dual regulatory modes from the perspectives of intercellular transmission and epitranscriptomic modification; (4) to summarize current research limitations and propose targeted experimental strategies and future research directions. This review fills the research gap caused by blurred boundaries between the two mechanisms in existing literature, and provides a standardized reference for subsequent mechanistic research and translational exploration of skin aging.

## 2 Criteria for identifying ncRNAs regulatory modes

As illustrated in Figure 1, ncRNAs regulate dermal fibroblast senescence via two mutually distinct mechanisms: direct regulation (left panel) and the ceRNA mechanism (right panel). The ceRNA mechanism relies on lncRNAs or circRNAs acting as molecular sponges in the cytoplasm to sequester miRNAs via miRNA response elements (MREs), thereby alleviating miRNA-induced repression of downstream target mRNAs and upregulating target gene expression. In contrast, direct regulation functions independently of miRNA sponging and encompasses three primary forms: (1) miRNA-mediated regulation, where miRNAs bind to the 3′untranslated region (3′UTR) of target mRNAs in the cytoplasm to induce mRNA degradation or translational repression; (2) lncRNA-mediated regulation, wherein cytoplasmic lncRNAs directly bind to signaling or scaffolding proteins to modulate their stability, activity, or subcellular localization, while nuclear lncRNAs interact with chromatin or DNA—typically by recruiting chromatin-modifying complexes or forming R-loops—to regulate gene transcription at the epigenetic level; and (3) circRNA-mediated regulation, in which predominantly cytoplasmic circRNAs interact with transcription factors, signaling molecules, or RNA-binding proteins to modulate their function or stability, generally without participating in direct DNA/chromatin regulation due to the lack of nuclear localization signals.

In this review, we collectively refer to the above-mentioned forms of miRNA-mRNA targeting and lncRNA/circRNA-protein/DNA binding as “direct regulation”. This is a functional classification term used solely to distinguish them from the ceRNA mechanism, highlighting their common feature: neither relies on miRNA-mediated ceRNA crosstalk. However, this classification does not imply that these forms share identical molecular principles or biochemical foundations. Specifically, miRNA-mRNA targeting primarily depends on sequence complementarity and RISC complex mediation, whereas lncRNA/circRNA-protein/DNA binding relies on interactions between RNA structural motifs and protein interaction domains. Table 1 summarizes the core criteria for distinguishing direct regulation from the ceRNA mechanism, including evidence of physical binding, functional site validation, ribonucleoprotein complex enrichment, and expression correlation, along with representative or foundational studies. Subsequent sections will systematically categorize and critically evaluate the existing literature based on these criteria.

**TABLE 1:** Comparison of core criteria for direct regulation and ceRNA mechanisms.

Assessment criteria	Direct regulation	ceRNA mechanism	References
core definitions	miRNA-mediated direct RNA-RNA targeting and lncRNAs/circRNAs-mediated direct binding to proteins or DNA/chromatin — neither relies on miRNA sponging	ncRNAs competitively bind to miRNAs via MREs, thereby indirectly lifting the miRNA’s suppression of the target mRNA	Gao et al. (2023), Kim et al. (2021), Zou et al. (2023)
physical binding evidence (required)	*In vivo*/in cell: RIP, CLIP-seq (PAR-CLIP, eCLIP, etc.) *In vitro*: EMSA, RNA pull-down. (Requires demonstration of direct complex formation between ncRNA and target proteins/nucleic acids)	RISC complex enrichment: AGO2-RIP experiments demonstrate that ncRNAs, miRNAs, and target mRNAs reside within the same silencing complex	Das et al. (2025), Donga et al. (2025), Martindale et al. (2020), Tsuji, 2023; Zou et al. (2023)
functional site validation (required)	miRNA: reporter gene assays comparing the 3′UTR of the target gene in the wild-type vs. seed sequence mutant lnc/circRNA: binding domain mutants for rescue assays to demonstrate the necessity of the interaction interface	MREs validation:Dual luciferase reporter assay. NcRNA fragments containing MREs suppress reporter gene expression; this suppression is enhanced by miRNA mimics and reversed by miRNA inhibitors; the effect disappears upon MRE mutation	Gao et al. (2023), Huang et al. (2025), Tang et al. (2023), Yan et al. (2023)
functional dependency	Knockdown/overexpression of ncRNAs directly affects target molecule levels and phenotypes; rescue assays require an intact binding interface	Bidirectional rescue: ncRNAs overexpression + co-expression of miRNA → reversal of phenotype. ncRNAs knockdown + miRNA inhibition →rescue of the phenotype	Lee et al. (2022), Tang et al. (2023)
expression correlation	miRNAs typically exhibit a negative correlation with target mRNAs (degradation/inhibition of translation)	ncRNA and target mRNA show a positive correlation, and this correlation depends on the expression level of the shared miRNA (dose-dependent effect)	Huang et al. (2025),Zhang et al. (2024c)
chromatin localization (chromatin-associated types only)	Techniques such as ChIRP-seq are used to identify genomic binding sites (promoters/enhancers)	Not applicable	Cozzolino et al. (2021)

Abbreviations: RIP, RNA, immunoprecipitation; CLIP-seq, Crosslinking and immunoprecipitation sequencing; PAR-CLIP, Photoactivatable-ribonucleoside-enhanced cross-linking and immunoprecipitation; eCLIP, Enhanced cross-linking and immunoprecipitation; EMSA, electrophoretic mobility shift assay; ChIRP-seq, Chromatin isolation by RNA, purification sequencing; 3′UTR, 3′untranslated region.

## 3 Direct regulatory mechanisms of ncRNAs in dermal fibroblast senescence

### 3.1 miRNA-mediated post-transcriptional regulation

miRNAs are a class of ncRNAs approximately 22 nucleotides in length that primarily regulate gene expression at the post-transcriptional level by binding to the 3’UTR of target mRNAs, thereby mediating target mRNAs degradation or inhibiting translation (Huang et al., 2025).

On the one hand, anti-aging miRNAs are downregulated under stress or aging conditions, thereby weakening the suppression of pro-aging pathways. In a D-galactose-induced senescence model, miR-208a-5p was shown to accelerate the aging process by inhibiting mitophagy through its targeting of Optic Atrophy 1 (OPA1) (Huang et al., 2025). In human dermal fibroblasts (HDFs) exposed to ultraviolet B (UVB) irradiation, miR-29b-3p expression was significantly reduced; functional rescue experiments indicated that miR-29b-3p directly binds to the 3′UTR of matrix metalloproteinase-2 (MMP-2), inhibiting MMP-2 expression, thereby reducing type I collagen degradation and promoting cell migration (Yan et al., 2023). Furthermore, downregulation of miR-146a has been observed in dermal fibroblasts from aged mice; its overexpression can improve the aging microenvironment by simultaneously targeting interleukin-1 receptor-associated kinase 1(IRAK1), TNF receptor-associated factor 6 (TRAF6), and NADPH Oxidase 4 (NOX4), thereby inhibiting both NF-κB-mediated chronic inflammation and nicotinamide adenine dinucleotide phosphate (NADPH) oxidase-dependent reactive oxygen species (ROS) accumulation (Zhang et al., 2024c).

On the other hand, senescence-promoting miRNAs are abnormally upregulated in models of replicative or photoaging, directly driving the aging phenotype. In dermal fibroblasts undergoing replicative senescence, miR-10a, miR-30c, and miR-451a were identified as synergistically upregulated; these three miRNAs target ephrin type-a receptor 4 (EPHA4), runt-related transcription factor 2 (RUNX2), and interleukin-6 receptor (IL6R), respectively, leading to increased senescence-associated beta-galactosidase (SA-β-gal) activity, enhanced secretion of SASP, and mitochondrial dysfunction (Lee et al., 2022). Reports indicate that UVB can induce the upregulation of miR-663a and miR-4706; together, they target the 3′UTR of Sirtuin 6 (SIRT6), suppressing SIRT6 expression and promoting DNA damage and ROS accumulation (Li et al., 2025). Studies have found that miR-100–3p accelerates photoaging by directly inhibiting ERBB receptor feedback inhibitor 1 (ERRFI1) and that epigenetic modifications may influence its targeting efficiency (Chen et al., 2024). In an H_2_O_2_/UVB stress model, hsa-miR-4535 was found to be upregulated; by binding to the 3′UTR of mothers against decapentaplegic homolog 4 (Smad4), it inhibits the transforming growth factor-beta (TGF-β)/Smad signaling pathway, reducing the synthesis of type I and type III collagen. Conversely, downregulation of hsa-miR-4535 restores Smad4 expression and alleviates collagen degradation (Lee et al., 2021). In a thermal injury model, miR-506–3p was found to be significantly upregulated; it inhibits TGFB1 expression by directly binding to the 3′UTR of TGF-β1, thereby impairing fibroblast proliferation, migration, and type I collagen synthesis; inhibition of miR-506–3p reverses this dysfunction (Yang et al., 2021).

### 3.2 Direct regulation of lncRNAs

lncRNAs are a class of ncRNAs longer than 200 nucleotides that participate in various biological processes, including transcription, post-transcriptional regulation, and epigenetic regulation of gene expression (Frediani et al., 2025). Given the scarcity of direct evidence in dermal fibroblasts, the lncRNA functions discussed in this section are largely inferred from other cellular systems or models. The precise mechanisms in dermal fibroblasts await rigorous experimental validation. In replicative and stress-induced aged HDFs, lncRNA PURPL was found to be upregulated in aged cells, and its silencing reduced p53 phosphorylation levels (Frediani et al., 2025). Although lncRNA PURPL has been reported to directly bind to the MYB binding protein 1A (MYBBP1A) in other cell lines (Li et al., 2017), this direct regulation has not yet been validated by RIP or RNA pull-down assays in dermal fibroblasts, and the specific mechanism by which it regulates p53 (direct binding or indirect regulation) requires further investigation.

### 3.3 Direct regulation of circRNAs

circRNAs are covalently closed circular RNA molecules formed by back-splicing of precursor mRNAs. Due to the absence of a 5′cap structure and a 3′poly-A tail, they are highly resistant to exonucleases and thus exhibit high stability, making them difficult to degrade (Zhang et al., 2022). This unique structural feature not only endows circRNAs with the potential to serve as biomarkers but also provides the foundation for them to perform diverse molecular functions. In addition to acting as miRNA sponges, circRNAs can exert key regulatory functions by directly interacting with proteins. Previous studies in models of muscular atrophy have demonstrated (Chen et al., 2023) that circTmeff1 can directly bind to tar DNA binding protein-43 (TDP-43), thereby alleviating muscle cell apoptosis and tissue degeneration. However, although successful precedents of circRNA-protein direct binding exist in other disease models, whether circRNAs exert their functions through direct protein interactions in dermal fibroblast senescence remains completely unknown. This direction urgently awaits exploration.

## 4 Indirect regulation via ceRNA networks

### 4.1 ceRNA axes regulating aging in dermal fibroblasts

To provide a visual summary of existing findings, Table 2 lists the major ceRNA axes involved in regulating dermal fibroblast senescence discussed in this section, along with their respective references. Notably, the senescence process of dermal fibroblasts is not determined by a single ceRNA axis in isolation but is coordinately regulated by multiple ceRNA axes. These axes may form complex regulatory networks by competitively binding the same miRNAs or converging on key signaling pathways. This multilayered regulatory mechanism ensures that cells can respond finely to environmental stress, thereby determining their senescence fate. Future studies should further elucidate the interactions among these axes and their hierarchical relationships within the aging network.

**TABLE 2:** Summary of key ceRNAs axes regulating senescence in dermal fibroblasts.

Aging model	ceRNAs	Change in expression	miRNA	Downstream target genes/Pathways	Biological effects of overexpression	References
Axes associated with replicative and natural aging	circ004463	↓	miR-23 b	CADM3, MAP4K4/AKT/ERK	Promotes cell proliferation and collagen synthesis, and helps combat skin aging	Zou et al. (2023)
	LincRNA-EPS	↓	miR-34a	CCND1	Promotes cell cycle progression and slows down aging	Zhang et al. (2024b)
	lncRNA H19	↓	miR-296–5p	IGF2/PI3K/AKT/mTOR	Maintain fibroblast homeostasis and slow down intrinsic aging	Tang et al. (2022)
Photoaging-related axes	lncRNA H19	↓	miR-138	SIRT1	Enhances antioxidant activity, inhibits MMPs, and promotes collagen synthesis	Gao et al. (2023)
	lncRNA PVT1	↓	miR-551b-3p	AQP3/ERK/p38 MAPK	Inhibits the expression of aging biomarkers, alleviates cell cycle arrest, and enhances cell vitality	Tang et al. (2023)
Axes associated with stress-induced and genetic senescence	circAMD1	↓	miR-27a-3p	collagen I	Promotes cell proliferation, restores collagen synthesis, and combats premature aging	Su et al. (2021)
	lncRNA TPT1-AS1	↓	miR-324–5p	CDK16	Promotes ECM synthesis and inhibits apoptosis	Luo et al. (2021)

#### 4.1.1 Axes associated with replicative and natural aging

In replicative senescence models, multiple ceRNA axes with well-defined functions have been identified (Table 2). In porcine dermal fibroblasts, highly expressed endogenous circ004463 can promote cell proliferation and collagen synthesis. Furthermore, in a naturally aged mouse model, exogenous overexpression of circ004463 was found to sequester miR-23b, upregulate Cell Adhesion Molecule 3 (CADM3) and Mitogen-Activated Protein Kinase Kinase Kinase Kinase 4 (MAP4K4), activate the protein kinase B (AKT)/extracellular signal-regulated kinase (ERK) signaling pathway, and thereby promote cell proliferation and collagen synthesis, reversing the aging-related degeneration of skin structure (Zou et al., 2023). This suggests that the circ004463/miR-23b/CADM3-MAP4K4 axis may be a universal regulatory pathway across species and aging models. In naturally aged HDFs, LincRNA-EPS expression is significantly downregulated; its overexpression can promote cell proliferation, while its silencing exacerbates the senescence phenotype (Zhang et al., 2024b). Mechanistically, LincRNA-EPS acts as a ceRNA to sequester miR-34a, relieving the latter’s repression of the key cell cycle gene Cyclin D1 (CCND1), thereby restoring cell cycle progression. The LincRNA-EPS/miR-34a/CCND1 axis is thus established as a classical anti-senescence pathway in HDFs. Additionally, the lncRNA H19/miR-296–5p/insulin-like growth factor 2 (IGF2) axis has been shown to play an important role in the same model (Tang et al., 2022). Downregulation of lncRNA H19 leads to upregulation of miR-296–5p, which in turn inhibits the expression of IGF2, weakens PI3K/AKT/mTOR signaling pathway activity, and reduces aquaporin 3 (AQP3) levels, ultimately triggering cell cycle arrest. Restoring lncRNA H19 expression can effectively alleviate the above phenotypes, indicating that this axis plays a central role in maintaining fibroblast homeostasis and delaying intrinsic aging.

#### 4.1.2 Photoaging-related axes

In UV-induced photoaging models, ceRNA networks also play a crucial protective (Table 2). In UVB-irradiated HDFs and mouse skin models (Gao et al., 2023), lncRNA H19 expression is downregulated; restoration of lncRNA H19 sequesters miR-138, thereby relieving its repression of Sirtuin 1 (SIRT1), enhancing antioxidant capacity, suppressing the MMP-1, MMP-3, and MMP-9 expression, and promoting type I collagen synthesis. The functional loop of the lncRNA H19/miR-138/SIRT1 axis has been validated by dual-luciferase reporter assays and functional rescue assays, establishing this ceRNA pathway as anti-photoaging. Additionally, in UVB-irradiated HDFs (Tang et al., 2023), lncRNA PVT1 expression is significantly decreased, accompanied by upregulation of miR-551b-3p and suppression of its target gene AQP3. LncRNA PVT1 acts as a sponge for miR-551b-3p to restore AQP3 expression and inhibit ERK/p38 MAPK pathway activity, thereby alleviating cell cycle arrest and enhancing cell viability, which establishes the lncRNA PVT1/miR-551b-3p/AQP3 axis as a defensive mechanism in photoaging.

#### 4.1.3 Axes associated with stress-induced and genetic senescence

In the context of atypical senescence, ceRNA mechanism also participate in stress responses and the regulation of genetic disorders (Table 2). In a model of genetic progeria, circAMD1 is significantly downregulated in P63-mutant dermal fibroblasts, leading to elevated miR-27a-3p activity and suppression of collagen synthesis pathways (Su et al., 2021). Functional rescue assays have demonstrated that overexpression of miR-27a-3p completely reverses the protective effect of circAMD1, strongly supporting the functional relevance of the circAMD1/miR-27a-3p axis in genetic cutaneous progeria. In dermal fibroblasts subjected to thermal injury (Luo et al., 2021), lncRNA TPT1-AS1 expression is downregulated, whereas miR-324-5p is upregulated and directly targets cyclin-dependent kinase 16 (CDK16) to suppress its expression, thereby reducing type I collagen and alpha-smooth muscle actin (α-SMA) synthesis and promoting apoptosis. Overexpression of lncRNA TPT1-AS1 reverses these effects, indicating that the lncRNA TPT1-AS1/miR-324-5p/CDK16 axis plays a protective role in the repair process following acute thermal stress.

## 5 Dynamic regulatory dimensions and network integration mechanisms

### 5.1 Dynamic dimensions of ncRNAs regulation

#### 5.1.1 Intercellular transmission

Exosomes serve not only as transport carriers for ncRNAs (Hajialiasgary Najafabadi et al., 2024) but also as a pivotal platform for the programmed deployment of dual regulatory modes among cells. Donor cells can selectively package ncRNAs with specific functional patterns, enabling them to execute pre-set regulatory logic in recipient cells.

For example, exosomes derived from bone marrow mesenchymal stem cells deliver miR-29b-3p to human dermal fibroblasts; this miRNA directly binds to the 3′UTR of MMP-2 mRNA, inhibits its expression, reduces type I collagen degradation, and delays skin aging (Yan et al., 2023). This represents a typical miRNA direct targeting mechanism. Subsequent studies further revealed the ceRNA paradigm of exosomal lncRNA delivery (Gao et al., 2023), in which exosomes from bone marrow mesenchymal stem cells deliver lncRNA H19 to UVB-damaged human dermal fibroblasts, upregulate SIRT1 expression by sponging miR-138, and thereby alleviate photoaging-induced cellular senescence. Recent studies have confirmed that exosomal delivery of circ0011129 is an effective anti-photoaging strategy. The delivered circ0011129 significantly downregulates the activity of the p53/p21/p16 senescence pathway and effectively restores the synthesis of type I collagen and elastin, thereby exerting a potent anti-aging effect (Zhang et al., 2022; Zhang et al., 2025). Although the functional output of this circRNA has been clearly established, its precise mode of action remains purely speculative due to a lack of direct experimental evidence confirming whether it acts as a ceRNA sponge or through direct regulation mechanisms. Similarly, lncRNA VIM-AS1 carried by exosomes from human umbilical cord mesenchymal stem cells can be taken up by dermal fibroblasts, promoting glycolysis and reducing ROS levels by activating the PPAR-γ pathway, accompanied by upregulated SIRT1 expression, ultimately leading to indirect inhibition of the p53/p16 pathway and alleviation of cellular senescence (Liang et al., 2025). However, this study was primarily based on phenotypic observations and did not clarify the specific molecular mode of action of lncRNA VIM-AS1 through biochemical validation; its precise downstream targets remain to be explored. These cases collectively highlight a central scientific question: which regulatory mode is activated when exosome-delivered ncRNAs enter recipient cells? Is this mode determined by the microenvironment of the recipient cell (e.g., stress status, expression profile of modifying enzymes)?

It is noteworthy that small extracellular vesicles (sEVs) secreted by dermal fibroblasts themselves play a dual role in skin homeostasis and aging, and their functions are highly dependent on the state of donor cells. Under pathological conditions, replicatively senescent fibroblasts secrete sEVs rich in miR-10a, miR-30c, and miR-451a (Lee et al., 2022). After being taken up by adjacent normal fibroblasts, these sEVs directly target and inhibit genes such as EPHA4, RUNX2, and IL6R through miRNAs, inducing an increase in the secretion of the SASP and mitochondrial dysfunction, thereby mediating paracrine senescence propagation. This study identified only the miRNA components in pro-senescent sEVs. In the future, if it is found that these sEVs simultaneously carry functional ncRNAs that can directly bind to signaling proteins, they may superimpose pro-senescent effects through non-miRNA mechanism, forming a multimodal regulatory network. Conversely, under physiological conditions, sEVs derived from healthy dermal fibroblasts effectively promote fibroblast proliferation, migration, collagen synthesis, and skin structural integrity by carrying active molecules such as miR-218 and integrin subunit beta like 1 (ITGBL1) (Zou et al., 2022), exerting a positive homeostatic maintenance effect. The above contrast clearly indicates that the functional output of sEVs is not inherent but is programmed by the microenvironment of donor cells.

Cross-species exosome systems further expand this dimension. Plant-derived exosome-like nanovesicles (OLELNVs) (Wang Z. et al., 2024) can efficiently deliver miR168a-5p to human dermal cells, accompanied by significant inhibition of the NF-κB signaling pathway. Bioinformatic analysis predicts that miR168a-5p targets key nodes within this pathway. However, this prediction awaits experimental validation. Whether its specific mechanism of action involves direct regulatory targeting or indirect modulation of the inflammatory microenvironment remains unknown. Although functional lncRNAs have not yet been detected in OLELNVs, if plant-derived lncRNAs are found in the future to directly bind conserved domain-containing proteins in human cells, this would establish a new paradigm of cross-kingdom direct regulation. In summary, exosomes not only serve as intercellular transport carriers for ncRNAs, but may also, through selective packaging of ncRNAs with specific functional modes, determine whether the regulatory logic in recipient cells operates via direct regulation or ceRNA sponging.

#### 5.1.2 Intramolecular regulation

The functions of ncRNAs are determined not only by their sequences but also dynamically regulated by epitranscriptomic modifications. N^6^-methyladenosine (m^6^A) is the most prevalent internal chemical modification in eukaryotic RNA and is widely involved in RNA processing, stability, and translational regulation (Cheng et al., 2022). During skin aging, environmental and metabolic stresses can dynamically reshape the epitranscriptome by altering the expression of m^6^A-modifying enzymes, thereby modulating key signaling pathways.

m^6^A modification can indirectly influence the strength of direct regulation-mediated functions of miRNAs by regulating their maturation process. In UVB-induced photoaging models of human dermal fibroblasts (Chen et al., 2024), the expression of Methyltransferase-like 14 (METTL14) is significantly downregulated, leading to reduced m^6^A modification on primary miR-100 (pri-miR-100). This reduction in modification impairs the binding efficiency between pri-miR-100 and DGCR8, inhibiting processing of pri-miR-100 into mature miR-100–3p. MiR-100–3p targets ERRFI1 mRNA through direct regulation and suppresses its translation; when miR-100–3p levels decrease, ERRFI1 protein expression increases, which subsequently inhibits the epidermal growth factor receptor (EGFR)-AKT signaling pathway and indirectly activates the p53/p21 pathway, ultimately promoting reduced collagen synthesis and the development of senescence phenotypes. Further studies have confirmed that overexpression of METTL14 restores miR-100–3p levels, suppresses ERRFI1, and significantly alleviates UVB-induced photoaging, revealing a photoprotective role of METTL14 mediated through m^6^A-dependent processing. On the other hand, advanced glycation end products (AGEs), as endogenous drivers of photoaging, can upregulate METTL14 expression and elevate global m^6^A modification levels. In AGEs-treated human dermal fibroblasts (Ouyang et al., 2022), METTL14 expression is markedly upregulated, resulting in increased global m^6^A modification. m^6^A epitranscriptome profiling reveals that differentially methylated mRNAs and lncRNAs are significantly enriched in inflammation-related pathways such as tumor necrosis factor (TNF) and interleukin-17 (IL-17) signaling, with multiple genes involved in NF-κB signaling regulation. Importantly, concurrent accumulation of AGEs, enhanced METTL14 protein expression, and elevated m^6^A levels have been observed in sun-exposed human skin, suggesting that the AGEs-METTL14-m^6^A axis may accelerate structural skin degeneration by driving chronic low-grade inflammation.

Collectively, these findings indicate that diverse environmental and metabolic stresses can reprogram the epitranscriptomic landscape of ncRNAs and their associated signaling transcripts by modulating m^6^A-modifying enzyme activity, thereby playing a critical regulatory role in the process of skin aging.

### 5.2 Network integration in dual mode

#### 5.2.1 Pathway convergence

**FIGURE 2:**
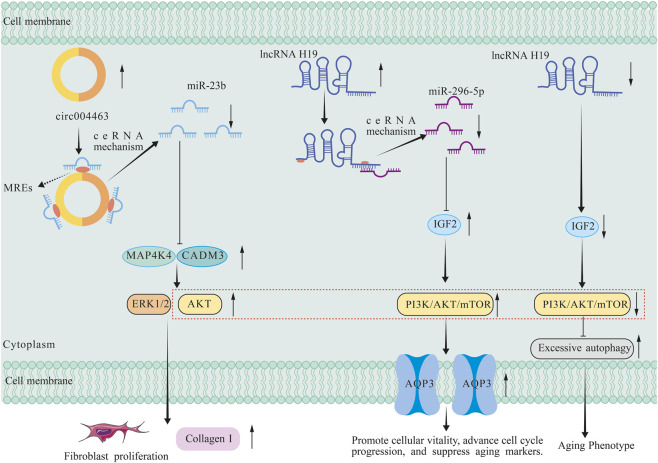
Multimodal synergistic activation network in the PI3K/AKT pathway. The lncRNA H19 and circ004463 target different nodes in the signaling pathway through ceRNA mechanisms or potential direct protein-protein interactions, thereby activating or inhibiting AKT signaling and exerting either pro-aging or anti-aging effects in the aging of dermal fibroblasts.

In the regulatory network of dermal fibroblast senescence, ncRNAs do not act in isolation; rather, they establish a multi-layered and dynamically plastic regulatory architecture within key signaling pathways through two core modes: direct protein interaction and ceRNA sponging (Figure 2). Among them, lncRNA H19 is a representative molecule that has been confirmed by multiple independent studies to possess context-dependent multimodal regulatory capabilities. In various models such as UVB photoaging, natural aging, and replicative senescence, lncRNA H19 is significantly downregulated, and its overexpression exerts anti-aging effects through distinct molecular mechanisms (Frediani et al., 2025; Gao et al., 2023; Tang et al., 2022). In naturally aged HDFs, lncRNA H19 expression is reduced; restoring lncRNA H19 expression upregulates IGF2 by sponging miR-296–5p, thereby activating PI3K/AKT signaling and delaying intrinsic senescence (Tang et al., 2022). Similarly, lncRNA H19 is markedly downregulated in both replicative and stress-induced premature senescence models. Knockdown of lncRNA H19 in young human dermal fibroblasts suppresses PI3K/AKT/mTOR pathway activity, induces autophagy, and triggers a typical senescence phenotype (Frediani et al., 2025). This finding reversely confirms the key role of lncRNA H19 in maintaining a youthful cellular state. Notably, this study focused on the functional consequences of lncRNA H19 and did not explore its upstream regulators or downstream mechanisms of action, nor did it detect the involvement of any miRNAs. Given that lncRNA H19 has a significant impact on the phosphorylation status of PI3K, AKT, and mTOR, it is hypothesized that its function in this context may involve direct binding to key proteins in signaling pathways rather than relying on the classic ceRNA mechanism. It should be emphasized that this hypothesis currently lacks direct experimental evidence and awaits validation through experiments such as RIP or pull-down assays. Overall, lncRNA H19 not only has dual modes of action but can also dynamically switch regulatory strategies according to the type of stress, becoming an adaptive hub in the aging network.

This multimodal input is manifested as the coordinated activation of multiple nodes in the PI3K/AKT pathway. On the one hand, circ004463 sponges miR-23b through the classic ceRNA mechanism, relieving its inhibitory effects on CADM3 and MAP4K4, thereby activating AKT and ERK signals, and promoting fibroblast proliferation and Collagen I synthesis (Zou et al., 2023). On the other hand, lncRNA H19 has also been confirmed to be a key positive regulator of this pathway: whether its absence in premature aging models leads to a decrease in AKT/mTOR phosphorylation (Frediani et al., 2025), or its restoration of pathway activity through the ceRNA mechanism in natural aging models (Tang et al., 2022), both indicate that lncRNA H19 plays an indispensable driving role in the PI3K/AKT/mTOR axis, and its mechanism may encompass both ceRNA and non-ceRNA modes. It can be seen that the PI3K/AKT pathway is doubly driven by circ004463 (an independent ceRNA axis) and lncRNA H19 (multiple regulatory modes). Although their target sites and specific mechanisms differ, they ultimately converge on AKT activation. This multi-level regulatory network jointly constructs a robust defense line against skin aging through a functional redundancy mechanism.

#### 5.2.2 Functional antagonism

**FIGURE 3:**
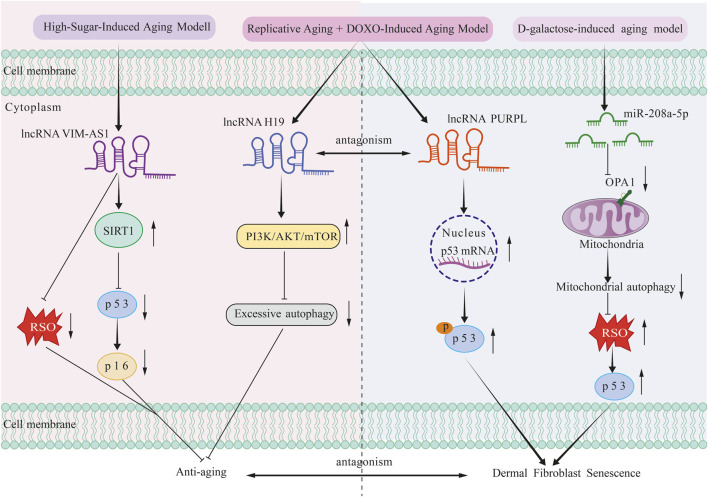
Functional antagonism network of ncRNAs in senescence regulation. Anti-aging ncRNAs (lncRNA H19 and lncRNA VIM-AS1) and pro-aging ncRNAs (lncRNA PURPL and miR-208a-5p) inhibit or activate p53 and related pathways, respectively, forming a dynamic regulatory network.

In the p53 pathway-dominated senescence regulatory network, multiple ncRNAs exert functionally antagonistic effects through distinct mechanisms, collectively maintaining dynamic equilibrium in cell fate decisions (Liang et al., 2025). On the one hand, certain ncRNAs act as brakes to suppress the p53 pathway and delay senescence. For example, lncRNA VIM-AS1 significantly inhibits key senescence markers such as p53 and p16 in high glucose-induced senescence models by upregulating SIRT1 expression and reducing ROS levels. On the other hand, other ncRNAs serve as accelerators that positively drive the p53 pathway. Representative examples include lncRNA PURPL, which is upregulated in multiple senescence models; knockdown experiments show that it reduces both p53 mRNA levels and ATM-mediated phosphorylation, indicating that lncRNA PURPL is a critical positive regulator of the p53 pathway. Although the specific mechanism has not been fully elucidated, we speculate that it may involve direct regulation or ceRNA function. However, this remains a hypothesis based on phenotypic observations and urgently requires experimental validation such as RIP or pull-down assays. (Frediani et al., 2025); and miR-208a-5p, which directly targets the mitochondrial fusion protein OPA1, inducing mitochondrial dysfunction and oxidative stress, thereby activating a p53-dependent senescence program (Huang et al., 2025). Notably, this antagonism manifests at the systems level as cross-pathway counterbalancing. In a study of the same senescence model, lncRNA H19 and lncRNA PURPL exhibited significantly opposite expression patterns (Frediani et al., 2025): lncRNA PURPL was upregulated to promote p53-mediated senescence, while lncRNA H19 downregulation led to attenuation of the pro-survival PI3K/AKT/mTOR signaling. Although they act on distinct signaling nodes (p53 versus PI3K/AKT), this reciprocal relationship reveals a deep functional antagonism within the senescence regulatory network: loss of lncRNA H19 weakens cellular anti-aging capacity, whereas gain of lncRNA PURPL enhances senescence-driving force. Collectively, these findings indicate that the p53 pathway does not operate in isolation but functions as an integrative hub that receives multimodal inputs from diverse ncRNAs—including both direct regulation and ceRNA mechanism. These inputs establish a fine-tuned antagonistic balance between pro-senescence and anti-senescence signals, thereby enabling precise regulation of cellular homeostasis (Figure 3).

## 6 Limitations and future perspectives

### 6.1 Direct regulation studies require urgent reinforcement

Current research on the direct molecular interaction mechanisms of ncRNAs in dermal fibroblast senescence exhibits significant imbalance, with substantial differences in research depth across different categories of ncRNAs. As shown in Table 3, at the level of direct RNA-RNA targeting, miRNAs have been relatively well-studied in terms of direct regulation, with multiple regulatory axes validated by dual-luciferase reporter assays (Chen et al., 2024; Huang et al., 2025; Yan et al., 2023; Zhang et al., 2024c). In contrast, direct regulation studies on lncRNAs are markedly insufficient. For example, lncRNA H19 affects PI3K/AKT/mTOR pathway activity, lncRNA PURPL regulates p53 phosphorylation (Frediani et al., 2025), and lncRNA VIM-AS1 upregulates SIRT1 while downregulating p53 (Liang et al., 2025). However, whether these lncRNAs function through ceRNA mechanism or direct protein binding in dermal fibroblasts remains unclear, and biochemical validation such as RIP or RNA pull-down assays is lacking. For circRNAs, direct regulation research is completely absent. Although functional circRNAs have been reported via ceRNA mechanism in the field of dermal fibroblast senescence, no study has yet investigated whether they directly bind to senescence-associated signaling proteins. Notably, successful precedents exist in other disease models (Chen et al., 2023), suggesting significant potential in this direction. In addition, although some studies have suggested a potential regulatory relationship between miR-93 and SIRT1 (Promjantuek et al., 2022), this conclusion lacks experimental support for direct binding in dermal fibroblasts, and even the description in the original literature contains questionable points. Therefore, whether it constitutes direct targeted regulation requires rigorous validation.

**TABLE 3:** Current research status of direct ncRNA regulatory mechanisms in dermal fibroblast senescence.

ncRNA type	Representative ncRNAs	Research status	Interaction targets/Mechanisms	References
miRNAs	miR-208a-5p	Relatively well-studied	Targets OPA1, inhibits mitophagy	Huang et al. (2025)
	miR-29b-3p		Binds to the 3′UTR of MMP-2, inhibits collagen degradation	Yan et al. (2023)
	miR-146a		Targets IRAK1/TRAF6/NOX4	Zhang et al. (2024c)
	miR-100–3p		Targets ERRFI1	Chen et al. (2024)
lncRNAs	lncRNA H19	Insufficiently studied/Evidence lacking	Regulates PI3K/AKT/mTOR pathway	Frediani et al. (2025)
	lncRNA PURPL		Regulates p53 pathway	
	lncRNA VIM-AS1		Regulates SIRT1/p53 axis	Liang et al. (2025)
circRNAs	—	Research gap, no reports	—	—

Only representative miRNAs, with validated direct targeting in dermal fibroblasts are listed. Additional miRNAs, discussed in Section 3.1 are not included due to space constraints.

In summary, future studies urgently need to systematically apply experimental techniques such as RIP, CLIP-seq, and RNA pull-down to validate direct regulation of the aforementioned ncRNAs, with particular emphasis on filling the research gap for circRNAs.

### 6.2 Insufficient *in vivo* validation and physiological relevance of the ceRNA hypothesis

Although studies on the ceRNA mechanism have established multiple seemingly complete functional regulatory axes in the field of dermal fibroblast aging (such as the lncRNA H19/miR-296–5p/IGF2 axis and lncRNA PVT1/miR-551b-3p/AQP3 axis (Tang et al., 2023; Tang et al., 2022), their physiological relevance and *in vivo* robustness still face severe challenges. The core of this challenge lies not only in the lack of experimental validation but also in the neglect of the physical prerequisites for the ceRNA mechanism to take effect—stoichiometry and the threshold effect. The ceRNA mechanism does not occur linearly; its effectiveness strictly depends on the molar concentration ratio among ceRNAs, miRNAs, and their target mRNAs. A derepression effect occurs only when the expression abundance of the ceRNA reaches a specific threshold sufficient to competitively bind the limited pool of miRNAs within the cell (Denzler et al., 2014). However, many of the aforementioned studies share a critical methodological flaw: overreliance on *in vitro* overexpression models. In high-dose transfection experiments, researchers artificially elevate ceRNA concentrations far beyond physiological levels, thereby forcibly satisfying the stoichiometric requirement and observing a pronounced “sponge effect”. This experimentally induced extreme condition often masks a crucial reality: under physiological conditions or in the microenvironment of natural aging, the absolute copy numbers of many candidate ceRNAs may be far below the threshold required to effectively compete with miRNAs, rendering them incapable of exerting the expected regulatory function *in vivo*.

Unfortunately, many reports, including the aforementioned ones, are still mainly based on the negative correlation of expression levels. In the existing literature, numerous reports infer the ceRNA mechanism solely based on the negative correlation of expression levels (i.e., downregulation of ceRNAs accompanied by downregulation of the target gene), while quantitative analysis of absolute copy numbers is lacking. For example, a study predicted in a UVA-induced photoaging model that miR-146a-5p regulates the inflammatory pathway through multiple lncRNAs (Lin et al., 2021); another report proposed that circRNA-406918 regulates cathepsin D (CTSD) through the ceRNA mechanism to affect the degradation of AGEs (Qu et al., 2023). Although these studies have confirmed phenotypic changes through functional experiments and provided bioinformatic predictions, they generally fail to address a key question: under physiological UVA damage conditions or in natural aging contexts, is the endogenous expression level of these lncRNAs/circRNAs sufficient to competitively bind to miR-146a-5p or other mediating miRNAs? Without key experimental evidence supporting the ceRNA mechanism, such as dual-luciferase reporter gene assays and AGO2-RIP, and without quantitative determination of the absolute molar ratios between molecules, these so-called ceRNA axes likely represent artifacts of *in vitro* high-expression conditions, and their real contributions in the complex skin microenvironment and at the whole-animal level are questionable. This neglect of stoichiometry is the core reason for the controversy surrounding the ceRNA mechanism. Many seemingly perfect regulatory networks *in vitro* become ineffective due to insufficient concentrations once subjected to the dilution effects and dynamic equilibrium of *in vivo* environments, which seriously limits the reliability of translating relevant findings into clinical applications.

To overcome the validation bottleneck of the ceRNA mechanism, future studies urgently need to shift from qualitative correlation to quantitative confirmation. It is recommended that in addition to conventional relative quantification, absolute quantification methods such as standard curve-based approaches or droplet digital PCR (ddPCR) be employed to precisely measure the copy numbers of ceRNAs, miRNAs, and their target mRNAs, thereby assessing whether they meet the stoichiometric threshold required for competitive binding. Concurrently, single molecule fluorescence *in situ* hybridization (smFISH) should be integrated to validate the subcellular colocalization of these three components at single cell resolution. Furthermore, physiological-level-gene edited animal models should be prioritized over *in vitro* overexpression systems to eliminate artifacts caused by non-physiological concentrations, thereby establishing ceRNA regulatory networks that are genuinely robust and physiologically relevant *in vivo*.

### 6.3 Misattribution of regulatory hierarchy and lack of systematic validation

Current ncRNA research commonly adopts a reductionist paradigm, whereby regulatory relationships are established by validating direct regulation between a single ncRNA and its target molecule. However, this fragmented strategy is prone to attribution errors in regulatory hierarchies in miRNA research. For example, miRNAs that are actually indirectly regulated by upstream ceRNAs may be misjudged as direct regulatory factors capable of functioning independently, or the complete ceRNA-miRNA-mRNA three-level regulatory chain may be fragmented into isolated direct targeting events of miRNAs on target mRNAs.

Taking miR-29b-3p as an example, existing studies have confirmed through dual-luciferase reporter assays and functional rescue experiments that this miRNA directly binds the 3′UTR of MMP-2 mRNA and suppresses collagen degradation (Yan et al., 2023). This conclusion is well supported at the molecular interaction level. However, under specific senescence conditions, if this miRNA is simultaneously subjected to sponge-mediated sequestration by lncRNA H19 or lncRNA PVT1, its inhibitory effect on MMP-2 is in fact the terminal output of a ceRNA regulatory network rather than an initiating driver event. Early studies, lacking systematic screening of upstream ncRNA regulators of miRNAs, likely misinterpreted such indirect regulation as autonomous direct action by the miRNA. The situation becomes even more complex in contexts of dual regulation. For example, one study reported that METTL14 regulates the processing and maturation of pri-miR-100 through m^6^A modification, representing a direct epitranscriptional regulatory mechanism (Chen et al., 2024); however, if miR-100–3p is concurrently sponged by a circRNA, this forms a multilevel cascade network integrating m^6^A mediated miRNA biogenesis, ceRNA-mediated miRNA sequestration, and direct miRNA-mediated repression of target genes. Focusing on any single layer alone results in hierarchical truncation of mechanistic interpretation.

Such misjudgments mainly stem from two limitations: (1) Technical temporality: A large number of miRNA studies were published before the popularization of the ceRNA hypothesis, lacking systematic upstream screening; (2) Validation inertia: The gold-standard status of AGO2-RIP and luciferase assays has reinforced the miRNA-centered perspective, neglecting parallel validation of upstream ncRNAs. To avoid hierarchical attribution errors, future research should establish a dual-evidence standard: While verifying the direct targeting of miRNAs (physical binding between miRNA and mRNA + functional necessity), it is necessary to clarify whether the miRNA is regulated by upstream ceRNAs through co-expression network analysis of ncRNA-seq and miRNA-seq, as well as rescue experiments of miRNA levels after overexpression/knockdown of lncRNAs/circRNAs. Only by excluding or integrating the upstream hierarchy can the true position of miRNAs be accurately defined, preventing the terminal effects of ceRNA mechanism from being misjudged as isolated direct regulation.

### 6.4 Scarcity of research on emerging ncRNAs and cell subpopulation specificity

In addition to classic miRNAs, lncRNAs, and circRNAs, emerging ncRNA categories such as PIWI-interacting RNAs (piRNAs), small nucleolar RNAs (snoRNAs), and transfer RNA-derived small RNAs (tsRNAs) have also been preliminarily confirmed to be involved in the regulation of dermal fibroblast aging, but their functional studies are extremely superficial. piRNAs are a class of small RNAs that mainly bind to the PIWI protein family. They can silence transposable elements through transcriptional or post-transcriptional mechanisms to maintain genome stability (Iwasaki et al., 2015). Recent studies have found that piRNAs exhibit significant differential expression in aged rat skin and participate in regulating fibroblast senescence, suggesting a potential anti-aging regulatory role (Zhao et al., 2025); however, their specific molecular targets and signaling pathways require further investigation. snoRNAs mainly function within the nucleolus and are essential for the processing and modification of ribosomal RNA (rRNA). Specific snoRNAs show altered expression in senescence-associated human dermal fibroblasts and can regulate cell proliferation (Yang et al., 2024), yet their precise functions and modes of action during the aging process remain unelucidated. TsRNAs are functional small RNAs generated by specific nuclease cleavage of mature tRNAs under stress conditions, primarily including tRNA derived fragments (tRFs) and stress-induced tiRNAs (Xu et al., 2025). Multiple studies consistently indicate that stress-induced tsRNAs are significantly upregulated in dermal fibroblasts upon UV irradiation and participate in photoaging by directly regulating target genes (Liu et al., 2025; Xu et al., 2025; Yao et al., 2025). These findings preliminarily suggest that piRNAs, snoRNAs, and tsRNAs may be involved in regulating senescence; however, their specific molecular mechanisms remain completely unknown. Related research is still at an early stage, and systematic regulatory networks have not yet been established, necessitating systematic experimental validation.

In addition, traditional transcriptome analysis regards dermal fibroblasts as a functionally homogeneous population, making it difficult to analyze their heterogeneous responses during the aging process. Recent studies have used single-cell RNA sequencing (scRNA-seq) and spatial transcriptomics to identify four functional subpopulations (APOD^+^, SFRP2^+^, POSTN^+^, PTGDS^+^) in human dermis, among which SFRP2^+^ reticular layer fibroblasts are the core executors of ECM homeostasis, and their function declines in the aging state (Yu et al., 2025). Although a large number of age-related lncRNAs have been identified based on population samples (Tsitsipatis et al., 2023), and the age-relatedness of circRNAs has been preliminarily explored, due to the lack of single-cell resolution, it is impossible to determine whether these ncRNAs are specifically enriched in a certain functional subpopulation. Currently, no study has depicted the expression profiles of ncRNAs in fibroblast subpopulations at the single-cell or spatial level, which seriously hinders the precise analysis of the cell-specificity of the aging regulatory network.

### 6.5 Bottlenecks from mechanism to translation

To overcome the aforementioned limitations, future research should pursue multidimensional efforts. At the mechanistic level, it is necessary to fill the research gap in direct regulation. Techniques such as RIP and CLIP-seq (Das et al., 2025; Martindale et al., 2020) should be utilized to verify the direct binding of ncRNAs to proteins and DNA. Additionally, the miRNA-target verification systems should be improved to balance the research depth of the two core regulatory modes. At the dimensional level, greater efforts should be made to explore emerging ncRNAs. Systematically identify their differential expression profiles during aging and elucidate the mechanisms through functional experiments. Meanwhile, fully leverage scRNA-seq and spatial transcriptomics techniques (Tsitsipatis et al., 2023; Yu et al., 2025) to map the expression profiles of ncRNAs in different fibroblast subpopulations, clarify the roles of specific ncRNAs in core functional subpopulations, and analyze the cell specificity of regulation. At the translational level, based on the well-defined functional ceRNA axes and direct regulation targets, precise intervention strategies such as exosome-mediated delivery of ncRNAs (Liang et al., 2025; Yan et al., 2023) and targeting key ncRNAs with small-molecule compounds (Luan et al., 2023) should be developed. Moreover, a comprehensive pre-clinical animal model and human clinical trial system should be established to accelerate the translation of basic research findings into precise anti-aging applications, providing solid theoretical and technical support for the mechanistic analysis and intervention of skin aging. Beyond the technical bottlenecks mentioned above, conceptual cognitive biases also constrain the precision of translational research. Although no published study has yet been explicitly corrected for mechanistic misclassification, this more likely reflects the current lack of unified validation standards in the field rather than the absence of misclassification risks. Numerous studies rely solely on bioinformatic predictions or single-dimensional validation without rigorous functional rescue experiments, which may lead to incorrect attribution of regulatory mechanisms. Such mechanistic misclassification will directly affect the efficacy of intervention strategies: for example, if direct regulation is erroneously assumed to be a ceRNA mechanism, therapies designed to target miRNA sponging capacity would be ineffective; conversely, overlooking the direct protein-binding functions of lncRNAs/circRNAs may result in missing critical therapeutic targets. Therefore, future research must establish a multi-dimensional validation system encompassing expression correlation, molecular interaction validation, and functional rescue to ensure the accuracy of mechanistic interpretation, thereby providing a reliable basis for the design of precision intervention strategies.

It is worth emphasizing that the dual modes of direct regulation and ceRNA-mediated regulation by ncRNAs in dermal fibroblast senescence are not mutually exclusive. Instead, they can coexist, synergize, and dynamically switch, demonstrating clear physiological feasibility and scientific rationality. The same ncRNA (e.g., lncRNA H19) may employ direct protein interaction or ceRNA sponging mechanism under different aging stages or stress conditions. Likewise, the same signaling pathway (e.g., PI3K/AKT, p53) can be co-regulated by both modes, forming a complementary, stable, and flexible regulatory network. Therefore, future research should not be confined to a single mechanism but should prioritize integrated validation of the dual modes, systematically dissecting their synergistic relationships, dynamic switching conditions, and physiological significance to provide a more comprehensive theoretical basis for precision anti-aging interventions.

## 7 Conclusion

This review systematically elucidates that ncRNAs construct a complex network regulating dermal fibroblast aging through two core paradigms: direct regulation and ceRNA-mediated competitive regulation. At the level of direct regulation, ncRNAs act as key effector molecules, directly interfering with transcription, translation, and signaling pathway activity. However, in this field, especially regarding the direct functions and biochemical verification of circRNAs, there are still significant gaps. In contrast, although the ceRNA mechanism has constructed multiple regulatory axes with functional closed loops and demonstrated translational potential, its *in vivo* physiological robustness is still limited by controversies over stoichiometric ratio thresholds and absolute copy numbers. Current research not only suffers from an imbalance in the exploration of the two core paradigms but also faces three major challenges: First, the functional landscapes of emerging non-classic ncRNAs (such as piRNAs and tsRNAs) remain largely uncharted. Second, there is a lack of analysis of the specific regulatory networks of fibroblast functional subpopulations at single-cell resolution. Third, the understanding of how exosome-mediated intercellular communication and epigenetic transcriptome modifications like m^6^A expand the spatiotemporal dimensions of ncRNA regulation is still insufficient. Looking ahead, breakthroughs in this field urgently require a shift from qualitative descriptions to quantitative confirmations. On the one hand, rigorous biochemical verification of direct regulation should be strengthened, and the stoichiometric prerequisites for ceRNA mechanism operation must be clarified. On the other hand, spatial transcriptomics, single-cell sequencing, and high-resolution imaging techniques should be integrated to systematically decode the dynamic roles of non-classic ncRNAs in specific cell subpopulations. Only in this way can a high-fidelity aging regulatory map be drawn, and the transition of precise anti-aging strategies based on ncRNA targets from proof-of-concept to clinical translation be truly promoted.
